# 4-(4-Nitro­phen­oxy)butanol

**DOI:** 10.1107/S1600536811007744

**Published:** 2011-03-05

**Authors:** Zareen Akhter, Vickie McKee, Muhammad Saif Ullah Khan, Bushra Iftikhar, Humaira M. Siddiqi

**Affiliations:** a Department of Chemistry, Quaid-i-Azam University, Islamabad 45320, Pakistan; b Chemistry Department, Loughborough University, Loughborough LE11 3TU, England

## Abstract

The crystal structure of the title compound, C_10_H_13_NO_4_, features inter­molecular O—H⋯O(nitro) hydrogen bonding, which links mol­ecules into supra­molecular chains running parallel to the *bc* diagonal. There is also π–π stacking between 4-nitro­phenyl groups, the inter­planar distance between the nitro­benzene rings being 3.472 (2) Å.

## Related literature

For background material on polymers containing flexible linkages, see: Chandrasekhar (2005 ▶); Patil *et al.* (2010 ▶); Schab-Balcerzak *et al.* (2002 ▶); Shahram Mehdipour-Ataei & Zigheimat (2007 ▶); Scholl *et al.* (2007 ▶); Shockravi *et al.* (2007 ▶). For studies on related compounds based on flexible monomers, see: Choi *et al.* (2004 ▶); Liu *et al.* (2008 ▶).
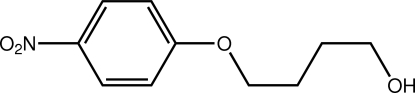


## Experimental

### 

#### Crystal data

C_10_H_13_NO_4_
*M* *_r_* = 211.21
Triclinic, 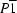

*a* = 4.7971 (6) Å
*b* = 10.6035 (13) Å
*c* = 11.2523 (14) Å
α = 117.521 (2)°
β = 92.451 (2)°
γ = 94.971 (2)°
*V* = 503.46 (11) Å^3^
*Z* = 2
Mo *K*α radiation
μ = 0.11 mm^−1^
*T* = 150 K
0.44 × 0.21 × 0.16 mm

#### Data collection

Bruker APEXII CCD diffractometer
Absorption correction: multi-scan (*SADABS*; Sheldrick, 2008*a* ▶) *T*_min_ = 0.954, *T*_max_ = 0.983
5772 measured reflections
2924 independent reflections
2224 reflections with *I* > 2σ(*I*)
*R*_int_ = 0.018

#### Refinement

*R*[*F* ^2^ > 2σ(*F* ^2^)] = 0.045
*wR*(*F* ^2^) = 0.139
*S* = 1.06
2924 reflections
139 parameters
H atoms treated by a mixture of independent and constrained refinement
Δρ_max_ = 0.33 e Å^−3^
Δρ_min_ = −0.24 e Å^−3^

### 

Data collection: *APEX2* (Bruker, 1998 ▶); cell refinement: *SAINT* (Bruker, 1998 ▶); data reduction: *SAINT*; program(s) used to solve structure: *SHELXS97* (Sheldrick, 2008*b* ▶); program(s) used to refine structure: *SHELXL97* (Sheldrick, 2008*b* ▶); molecular graphics: *SHELXTL* (Sheldrick, 2008*b* ▶); software used to prepare material for publication: *SHELXTL*.

## Supplementary Material

Crystal structure: contains datablocks I, global. DOI: 10.1107/S1600536811007744/tk2724sup1.cif

Structure factors: contains datablocks I. DOI: 10.1107/S1600536811007744/tk2724Isup2.hkl

Additional supplementary materials:  crystallographic information; 3D view; checkCIF report

## Figures and Tables

**Table 1:** Hydrogen-bond geometry (Å, °)

*D*—H⋯*A*	*D*—H	H⋯*A*	*D*⋯*A*	*D*—H⋯*A*
O4—H4⋯O3^i^	0.80 (3)	2.10 (2)	2.8808 (14)	163 (2)

Symmetry code: (i) 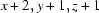.

**Fig. 1.**
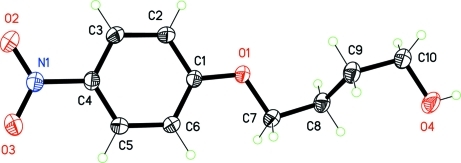
Perspective view of the molecule, showing ellipsoids at the 50% probability level. Hydrogen atoms are shown as arbitrary spheres.

**Fig. 2.**
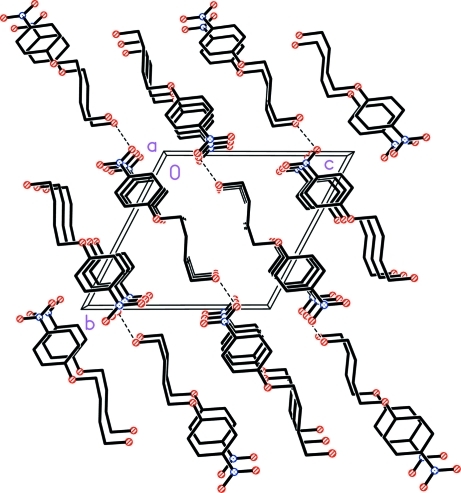
Packing diagram viewed down the a axis. Hydrogen atoms have been omitted and the dashed line represent O—H···O hydrogen bonds.

## supplementary crystallographic information

### Comment

Polymers are an important class of materials which have either supplemented or replaced conventional substances such as wood, stone, metal, glass and ceramics in modern technological applications (Chandrasekhar, 2005). Therefore, considerable research in recent years has focused upon producing novel polymeric materials with a better balance of physical and chemical properties (Shockravi *et al.*, 2007). Various flexible linkages such as the ether moiety (Patil *et al.*, 2010) and methylene spacers (Scholl *et al.*, 2007) can be introduced into the polymer backbone in order to improve their properties. The incorporation of an aryl-ether moiety is believed to impart enhanced solubility and processability to the polymers while maintaining their toughness (Shahram Mehdipour-Ataei & Zigheimat, 2007). On the other hand, the inclusion of aliphatic methylene spacers in the macrochain increases the degree of freedom by reducing the segmental barrier and effectively disrupts intermolecular interactions (Schab-Balcerzak *et al.*, 2002). Thus, the final polymer prepared from the monomers containing flexible linkages not only exhibits an enhancement in its processability but also shows an improvement in its performance as these flexible linkages also bestow mesogenic (Choi *et al.*, 2004) and optical properties (Liu *et al.*, 2008) to the resulting polymeric materials. The title compound, (I), Fig. 1, is a nitro-alcohol precursor with built-in methylene spacers along with aryl-ether moiety, which was prepared as part of our quest to design and synthesize structurally modified monomers for processable high performance polymers.

The alcohol group is H-bonded to the nitro group of a neighbouring molecule, Table 1. These link molecules into supramolecular chains running along the *bc* diagonal, Fig. 2. There are π-π interactions between the chains; the interplanar distance between the nitrobenzene rings is 3.472 (2) Å (symmetry operation: *x* - 1, *y*, *z*).

### Experimental

The title compound (I) was synthesized by Williamson's etherification of 1,4-butane diol and *p*-nitrochlorobenzene. A three-necked round bottom flask equipped with reflux condenser, thermometer and nitrogen inlet was charged with a suspension of 1,4-butane diol (1.69 ml; 19.1 mmol) and anhydrous potassium carbonate (2.65 g; 19.1 mmol) in dimethylformamide (40 ml) and stirred for 30 min. The resulting mixture was heated to 383–393 K for 6 h. The reaction mixture was poured into 500 ml of chilled water, cooled to room temperature and the crude product was filtered as a light-yellow solid mass. The product was then washed thoroughly with water, dissolved in ethanol and set aside for crystallization. Yield 79%, M.pt. 344 K.

### Refinement

H atoms were placed in calculated positions using a riding model with C—H distances constrained to 0.95 and 0.99 Å for aryl and methylene groups, respectively, and with *U*_iso_(H)=1.2 *U*_eq_(C). The hydrogen bonded to oxygen was located from difference maps; the coordinates were refined freely with*U*_iso_(H)=1.5 *U*_eq_(O).

### Crystal data

**Table:**

C_10_H_13_NO_4_	*Z* = 2
*M**_r_* = 211.21	*F*(000) = 224
Triclinic, *P*1	*D*_x_ = 1.393 Mg m^−^^3^
Hall symbol: -P 1	Melting point: 416 K
*a* = 4.7971 (6) Å	Mo *K*α radiation, λ = 0.71073 Å
*b* = 10.6035 (13) Å	Cell parameters from 1750 reflections
*c* = 11.2523 (14) Å	θ = 3.6–30.2°
α = 117.521 (2)°	µ = 0.11 mm^−^^1^
β = 92.451 (2)°	*T* = 150 K
γ = 94.971 (2)°	Block, yellow
*V* = 503.46 (11) Å^3^	0.44 × 0.21 × 0.16 mm

### Data collection

**Table:**

Bruker APEXII CCD diffractometer	2924 independent reflections
Radiation source: fine-focus sealed tube	2224 reflections with *I* > 2σ(*I*)
graphite	*R*_int_ = 0.018
φ and ω scans	θ_max_ = 30.4°, θ_min_ = 2.1°
Absorption correction: multi-scan (*SADABS*; Sheldrick, 2008*a*)	*h* = −6→6
*T*_min_ = 0.954, *T*_max_ = 0.983	*k* = −14→14
5772 measured reflections	*l* = −15→16

### Refinement

**Table:**

Refinement on *F*^2^	Primary atom site location: structure-invariant direct methods
Least-squares matrix: full	Secondary atom site location: difference Fourier map
*R*[*F*^2^ > 2σ(*F*^2^)] = 0.045	Hydrogen site location: inferred from neighbouring sites
*wR*(*F*^2^) = 0.139	H atoms treated by a mixture of independent and constrained refinement
*S* = 1.06	*w* = 1/[σ^2^(*F*_o_^2^) + (0.0693*P*)^2^ + 0.098*P*] where *P* = (*F*_o_^2^ + 2*F*_c_^2^)/3
2924 reflections	(Δ/σ)_max_ < 0.001
139 parameters	Δρ_max_ = 0.33 e Å^−^^3^
0 restraints	Δρ_min_ = −0.24 e Å^−^^3^

### Special details

**Table:**

Geometry. All e.s.d.'s (except the e.s.d. in the dihedral angle between two l.s. planes) are estimated using the full covariance matrix. The cell e.s.d.'s are taken into account individually in the estimation of e.s.d.'s in distances, angles and torsion angles; correlations between e.s.d.'s in cell parameters are only used when they are defined by crystal symmetry. An approximate (isotropic) treatment of cell e.s.d.'s is used for estimating e.s.d.'s involving l.s. planes.
Refinement. Refinement of *F*^2^ against ALL reflections. The weighted *R*-factor *wR* and goodness of fit *S* are based on *F*^2^, conventional *R*-factors *R* are based on *F*, with *F* set to zero for negative *F*^2^. The threshold expression of *F*^2^ > σ(*F*^2^) is used only for calculating *R*-factors(gt) *etc*. and is not relevant to the choice of reflections for refinement. *R*-factors based on *F*^2^ are statistically about twice as large as those based on *F*, and *R*- factors based on ALL data will be even larger.

### Fractional atomic coordinates and isotropic or equivalent isotropic displacement parameters (Å^2^)

**Table:**

	*x*	*y*	*z*	*U*_iso_*/*U*_eq_	
O1	0.44295 (19)	0.42787 (10)	0.15618 (9)	0.0266 (2)	
C1	0.2302 (3)	0.33362 (13)	0.06879 (12)	0.0218 (2)	
C2	0.1670 (3)	0.34735 (14)	−0.04730 (13)	0.0263 (3)	
H2	0.2744	0.4178	−0.0617	0.032*	
C3	−0.0513 (3)	0.25860 (14)	−0.14057 (13)	0.0265 (3)	
H3	−0.0961	0.2678	−0.2191	0.032*	
C4	−0.2045 (3)	0.15560 (13)	−0.11787 (12)	0.0223 (3)	
N1	−0.4365 (2)	0.06242 (11)	−0.21523 (11)	0.0252 (2)	
O2	−0.4898 (2)	0.07525 (11)	−0.31628 (10)	0.0348 (3)	
O3	−0.5727 (2)	−0.02676 (11)	−0.19184 (10)	0.0371 (3)	
C5	−0.1435 (3)	0.13965 (13)	−0.00455 (12)	0.0240 (3)	
H5	−0.2508	0.0684	0.0087	0.029*	
C6	0.0756 (3)	0.22859 (13)	0.08947 (12)	0.0240 (3)	
H6	0.1203	0.2182	0.1673	0.029*	
C7	0.5221 (3)	0.41461 (14)	0.27521 (13)	0.0261 (3)	
H7A	0.5888	0.3204	0.2494	0.031*	
H7B	0.3588	0.4228	0.3281	0.031*	
C8	0.7545 (3)	0.53402 (14)	0.35747 (13)	0.0265 (3)	
H8A	0.9078	0.5294	0.3000	0.032*	
H8B	0.8316	0.5188	0.4323	0.032*	
C9	0.6588 (3)	0.68272 (14)	0.41563 (13)	0.0281 (3)	
H9A	0.5873	0.6991	0.3407	0.034*	
H9B	0.5015	0.6865	0.4709	0.034*	
C10	0.8899 (3)	0.80179 (15)	0.50147 (13)	0.0298 (3)	
H10A	1.0544	0.7956	0.4497	0.036*	
H10B	0.8225	0.8961	0.5287	0.036*	
O4	0.9673 (3)	0.78689 (13)	0.61746 (11)	0.0442 (3)	
H4	1.096 (5)	0.847 (3)	0.661 (2)	0.066*	

### Atomic displacement parameters (Å^2^)

**Table:**

	*U*^11^	*U*^22^	*U*^33^	*U*^12^	*U*^13^	*U*^23^
O1	0.0287 (5)	0.0279 (5)	0.0219 (4)	−0.0073 (4)	−0.0059 (3)	0.0129 (4)
C1	0.0223 (6)	0.0224 (6)	0.0189 (5)	−0.0004 (4)	−0.0003 (4)	0.0088 (5)
C2	0.0293 (6)	0.0286 (6)	0.0235 (6)	−0.0036 (5)	0.0000 (5)	0.0156 (5)
C3	0.0294 (6)	0.0309 (6)	0.0214 (6)	−0.0016 (5)	−0.0011 (5)	0.0154 (5)
C4	0.0216 (6)	0.0225 (6)	0.0198 (5)	0.0002 (4)	−0.0005 (4)	0.0081 (5)
N1	0.0248 (5)	0.0258 (5)	0.0226 (5)	0.0000 (4)	−0.0013 (4)	0.0101 (4)
O2	0.0382 (6)	0.0388 (6)	0.0266 (5)	−0.0028 (4)	−0.0088 (4)	0.0170 (4)
O3	0.0369 (6)	0.0384 (6)	0.0329 (5)	−0.0153 (4)	−0.0073 (4)	0.0183 (5)
C5	0.0263 (6)	0.0244 (6)	0.0226 (6)	−0.0017 (5)	0.0005 (5)	0.0129 (5)
C6	0.0271 (6)	0.0255 (6)	0.0207 (5)	−0.0002 (5)	−0.0006 (5)	0.0128 (5)
C7	0.0272 (6)	0.0287 (6)	0.0226 (6)	0.0002 (5)	−0.0032 (5)	0.0133 (5)
C8	0.0225 (6)	0.0293 (6)	0.0245 (6)	0.0016 (5)	−0.0042 (5)	0.0107 (5)
C9	0.0261 (6)	0.0289 (7)	0.0243 (6)	0.0026 (5)	−0.0036 (5)	0.0088 (5)
C10	0.0339 (7)	0.0294 (7)	0.0223 (6)	−0.0018 (5)	−0.0016 (5)	0.0101 (5)
O4	0.0490 (7)	0.0521 (7)	0.0263 (5)	−0.0254 (5)	−0.0147 (5)	0.0208 (5)

### Geometric parameters (Å, °)

**Table:**

O1—C1	1.3551 (14)	C6—H6	0.9500
O1—C7	1.4484 (14)	C7—C8	1.5120 (17)
C1—C6	1.3984 (17)	C7—H7A	0.9900
C1—C2	1.4033 (16)	C7—H7B	0.9900
C2—C3	1.3805 (18)	C8—C9	1.5240 (19)
C2—H2	0.9500	C8—H8A	0.9900
C3—C4	1.3903 (17)	C8—H8B	0.9900
C3—H3	0.9500	C9—C10	1.5149 (18)
C4—C5	1.3842 (16)	C9—H9A	0.9900
C4—N1	1.4559 (15)	C9—H9B	0.9900
N1—O2	1.2252 (14)	C10—O4	1.4233 (17)
N1—O3	1.2361 (14)	C10—H10A	0.9900
C5—C6	1.3872 (17)	C10—H10B	0.9900
C5—H5	0.9500	O4—H4	0.80 (3)
			
C1—O1—C7	117.77 (10)	C8—C7—H7A	110.2
O1—C1—C6	123.97 (11)	O1—C7—H7B	110.2
O1—C1—C2	115.82 (11)	C8—C7—H7B	110.2
C6—C1—C2	120.20 (11)	H7A—C7—H7B	108.5
C3—C2—C1	120.07 (11)	C7—C8—C9	113.49 (11)
C3—C2—H2	120.0	C7—C8—H8A	108.9
C1—C2—H2	120.0	C9—C8—H8A	108.9
C2—C3—C4	118.99 (11)	C7—C8—H8B	108.9
C2—C3—H3	120.5	C9—C8—H8B	108.9
C4—C3—H3	120.5	H8A—C8—H8B	107.7
C5—C4—C3	121.74 (11)	C10—C9—C8	113.36 (11)
C5—C4—N1	118.96 (11)	C10—C9—H9A	108.9
C3—C4—N1	119.30 (11)	C8—C9—H9A	108.9
O2—N1—O3	122.62 (11)	C10—C9—H9B	108.9
O2—N1—C4	119.22 (10)	C8—C9—H9B	108.9
O3—N1—C4	118.16 (10)	H9A—C9—H9B	107.7
C4—C5—C6	119.48 (11)	O4—C10—C9	108.37 (11)
C4—C5—H5	120.3	O4—C10—H10A	110.0
C6—C5—H5	120.3	C9—C10—H10A	110.0
C5—C6—C1	119.51 (11)	O4—C10—H10B	110.0
C5—C6—H6	120.2	C9—C10—H10B	110.0
C1—C6—H6	120.2	H10A—C10—H10B	108.4
O1—C7—C8	107.39 (10)	C10—O4—H4	108.2 (17)
O1—C7—H7A	110.2		

### Hydrogen-bond geometry (Å, °)

**Table:**

*D*—H···*A*	*D*—H	H···*A*	*D*···*A*	*D*—H···*A*
O4—H4···O3^i^	0.80 (3)	2.10 (2)	2.8808 (14)	163 (2)

Symmetry codes: (i) *x*+2, *y*+1, *z*+1.
